# Cancer metastasis: molecular mechanisms and therapeutic interventions

**DOI:** 10.1186/s43556-025-00261-y

**Published:** 2025-04-07

**Authors:** Xiaofeng Dai, Ming Xi, Jitian Li

**Affiliations:** 1 National Local Joint Engineering Research Center for Precision Surgery & Regenerative Medicine, Shaanxi Provincial Center for Regenerative Medicine and Surgical Engineering, First Affiliated Hospital of Xi’an Jiaotong University, Xi’an, 710061 People’s Republic of China; 2 Molecular Biology Lab, Henan Luoyang Orthopedic Hospital (Henan Provincial Orthopedic Hospital), Henan Province, Zhengzhou, 450000 China

**Keywords:** Cancer, Metastasis organotropism, Onco-therapeutics, Oxidative stress, Metabolism, Cold atmospheric plasma

## Abstract

The metastatic cascade is a complicated process where cancer cells travel across multiple organs distant from their primary site of onset. Despite the wide acceptance of the ‘seed and soil’ theory, mechanisms driving metastasis organotropism remain mystery. Using breast cancer of different subtypes as the disease model, we characterized the ‘metastatic profile of cancer cells’ and the ‘redox status of the organ microenvironment’ as the primary determinants of cancer metastasis organotropism. Mechanically, we identified a positive correlation between cancer metabolic plasticity and stemness, and proposed oxidative stress as the selection power of cancer cells succeeding the metastasis cascade. Therapeutically, we proposed the use of pro-oxidative therapeutics in ablating cancer cells taking advantages of this fragile moment during metastasis. We comprehensively reviewed current pro-oxidative strategies for treating cancers that cover the first line chemo- and radio-therapies, approaches relying on naturally existing power including magnetic field, electric field, light and sound, nanoparticle-based anti-cancer composites obtained through artificial design, as well as cold atmospheric plasma as an innovative pro-oxidative multi-modal modality. We discussed possible combinations of pro-oxidative approaches with existing therapeutics in oncology prior to the forecast of future research directions. This paper identified the fundamental mechanics driving metastasis organotropism and proposed intervention strategies accordingly. Insights provided here may offer clues for the design of innovative solutions that may open a new paradigm for cancer treatment.

## Introduction

Under the long-term synergistic stimulus of various internal and external tumor-causing factors, cells loose the redox homeostasis [1, 2], leading to uncontrollable proliferation and the onset of carcinogenesis. Some transformed cells gain the metastatic features, explaining over 90% cancer-related mortality [3]. Metastasis refers to the process where transformed cells are disseminated from the site of primary initiation to different location(s) of the body. A complicated cascade is involved during metastasis including, e.g., invasion, intravasation, circulation, extravasation, and colonization. The organ to which cancer cells can metastasize is non-random, with the process being known as ‘organotropism’. For example, prostate cancers preferably colonize in the bone, uveal melanomas typically relapse to the liver, and breast cancer of different subtypes favor distinct organs to metastasize that include bone, liver, brain, and lung [4]. Transformed cells need to decide ‘whether to grow or go’ [5, 6] and ‘where to go’ for sustained survival, as proliferation and migration represent two mutually exclusive destinations as a result of competitive use of shared cellular resources [7]. Accumulated evidence has suggested that organotropism is regulated by multiple factors such as cancer stemness [8] and tumor matrix stiffness [7], implicating clues for effective cancer metastasis prognosis, prevention and therapeutics.

Breast cancer, remaining as the primary cause of female cancer deaths, is a heterogeneous disease that can be roughly divided into luminal A, luminal B, human epidermal growth factor receptor 2-positive (HER2 +), and triple negative breast cancer (TNBC) subtypes according to their biological characteristics and molecular markers [9–11]. Luminal A and B subtypes are over-represented with estrogen receptor (ER); HER2 + breast tumors are featured with high levels of HER2; TNBCs lack the expression of ER, progesterone receptor (PR), HER2 and thus are irresponsive to hormonal or targeted therapies such as Tamoxifen [12] and Herceptin [13]. Importantly, TNBCs are known to possess large percentages of cancer stem cells (CSCs), and are therefore more prone to develop metastasis and difficult to treat. Despite such distinct clinical manifestations, breast cancers of different subtypes display different metastatic tendencies. For instance, luminal A/B breast cancers are inclined to develop bone [14] and liver [15] metastasis, and HER2 + and TNBCs prefer to metastasize to visceral organs such as brain [16] and lung [8, 17–19]. Such distinct metastatic profiles of tumors originated from the same organ offer us an ideal disease model for investigating factors influencing or driving tumor metastasis organotropism.

The aim of this review is to aid in the effective prognosis, prevention and therapeutics of cancer metastasis for, hopefully, reduced cancer-associated mortality. Using breast cancers of different subtypes as the disease model, this review gives an overview on metastasis organotropism, characterizes critical factors defining cancer metastasis organotropism, identifies primary mechanisms driving the vulnerability of cancer cells to oxidative stress during metastasis, introduces current and emerging pro-oxidative onco-therapeutic modalities, discusses possible strategies for combining pro-oxidative approaches with existing therapeutics, and concludes this paper with future trends forecasted. 

## Metastasis organotropism

### Historical development of the ‘seed and soil’ theory

**Fig. 1:**
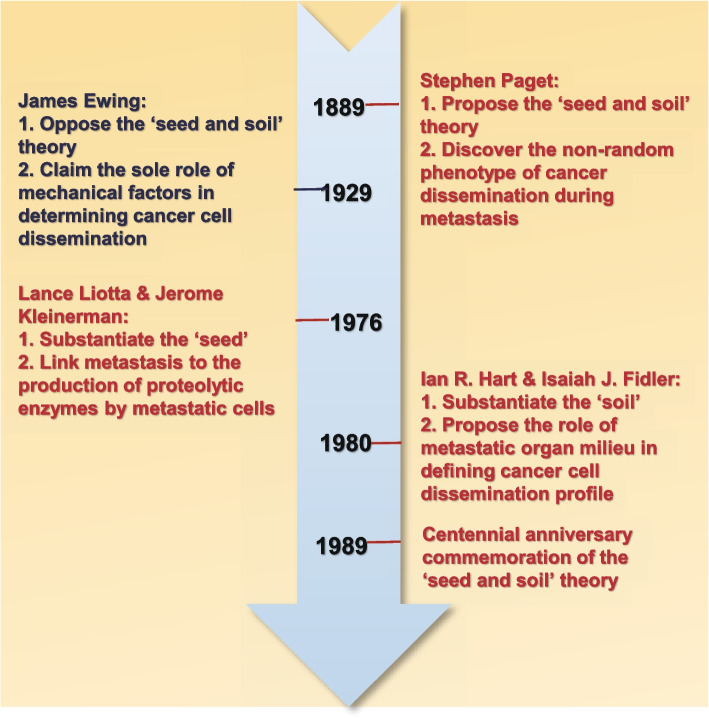
Historical development of the ‘seed and soil’ theory. Stephen Paget proposed the ‘seed and soil’ theory in 1889 to explain the non-random dissemination profile of cancer cells during metastasis he observed. James Ewing challenged this theory by claiming that cancer cell organotropism was solely influenced by mechanical factors. In 1970s, Lance Liotta & Jerome Kleinerman linked metastasis to the metastatic cell produced proteolytic enzymes, and Ian R. Hart & Isaiah J. Filder proposed the role of metastatic organ milieu in defining cancer cell dissemination profile that substantiated the ‘seed and soil’ theory from the ‘seed’ and ‘soil’ points respectively. In 1989, the ‘seed and soil’ theory was commemorated in its centennial anniversary

The ‘seed and soil’ theory was proposed initially by Stephen Paget to understand the non-random pattern of metastasis in 1889 [20]. By scrutinizing over 900 autopsy records of patients carrying different types of primary cancers, Stephen Paget was astonished in finding a discrepant pattern between the relative blood supply and the metastatic frequency of cancer cells to certain organs, i.e., there may exist a non-random distribution of metastatic cells to bones and visceral organs. Therefore, Stephen Paget challenged the prevailing view at that time in proposing the ‘seed and soil’ theory. This theory stated that the destination of metastatic cancer cells was not by chance but rather determined by cancer cells and the milieu of the organs; by considering tumor cells as the ‘seed’ and the milieu of certain organs as the ‘soil’, metastasis could only occur when the seed and the soil were compatible. However, Stephen Paget did not discuss in detail possible factors determining such a compatibility (Fig. 1).

The ‘seed and soil’ theory was challenged by James Ewing in 1929, who believed that metastatic dissemination of cancer cells, though not completely by chance, was dictated by mechanical factors such as the anatomical structure of the vascular system [21]. This view for the first time substantiated the ‘seed and soil’ theory in proposing possible influential factors on the compatibility between cancer cells and the colonized organ, but also negated the ‘seed and soil’ theory in attributing the metastasis organotropism purely to anatomical differences or, in other words, by chance (Fig. 1).

The viewpoint proposed by James Ewing prevailed for several decades until 1970s when Lance Liotta and Jerome Kleinerman characterized the association between cancer metastasis organotropism and the production of proteolytic enzymes by metastatic cancer cells [22]. Also, Ian R. Hart and Isaiah J. Fidler found that besides mechanical attract of traveling tumor cells in the capillary bed of distant organs, specific cells residing the metastatic organs played essential roles in the subsequent cancer cell proliferation in the secondary lesions during the colonization process using B16 melanoma as the cancer model [23]. Such viewpoints were supported by Leonard Weiss [24] and Everett V. Sugarbaker [25], who concluded that distant organ metastasis was site specific for a given type of cancers that could not be solely explained by anatomical or mechanical factors such as efferent venous circulation or lymphatic drainage to regional lymph nodes, after reviewing the clinical data of different human cancers regarding their site preferences of metastasis (Fig. 1).

After a century’s struggle and exploration on the possible existence of cancer metastasis organotropism, the ‘seed and soil’ theory was brought back to our horizon in 1989 when a symposium commemorated the centennial anniversary of Stephen Paget’s ‘seed and soil’ hypothesis. Ever since then, consecutive efforts have been devoted to update and substantiate the content and connotation of the ‘seed and soil’ theory with our incremental understandings on the pathogenesis and molecular features of cancers [4, 26]. The current beliefs on cancer metastasis organotropism contain the following three principles. First, primary and metastatic tumor lesions both contain cancer cells and host cells which are biologically heterogeneous. Primary components of the host cells include, e.g., epithelial cells, endothelial cells, fibroblasts and infiltrating leukocytes. Neoplasms contain genetically, epigenetically and phenotypically distinct cohorts of transformed cells, each of which have the potential to complete certain but not all steps in the metastatic process. Second, each of the five steps in the metastasis cascade (i.e., invasion, intravasation, circulation, extravasation, colonization) is selective for cells capable of surviving the stress under each particular circumstance, and metastasis can be originated from single cell expansion. To successfully pass through the whole metastasis cascade, metastatic cancer cells (the ‘seed’) must possess or evolve multiple features to be able to undertake a variety of tasks [27, 28], as the metastasis process may be blocked at diversified stages such as loss of the ability of progressive growth, vascularization and invasion, detachment from the cell cluster, embolization, susceptibility to the attack of the immune system, and the inability of usurping resources from the environment of the secondary loci. Third, a given cluster of travelling cancer cells can only surpass the dormant stage and regain uncontrolled proliferation in specific organs. The biological characteristics of the microenvironments of different metastatic organs (the ‘soil’) differ that are deterministic on the types of cancer cells capable of establishing colonization there. In other words, the outcome of metastasis depends on whether a homeostatic crosstalk can be developed between the metastasizing cells and the host environment.

### Breast cancer metastasis organotropism

**Fig. 2:**
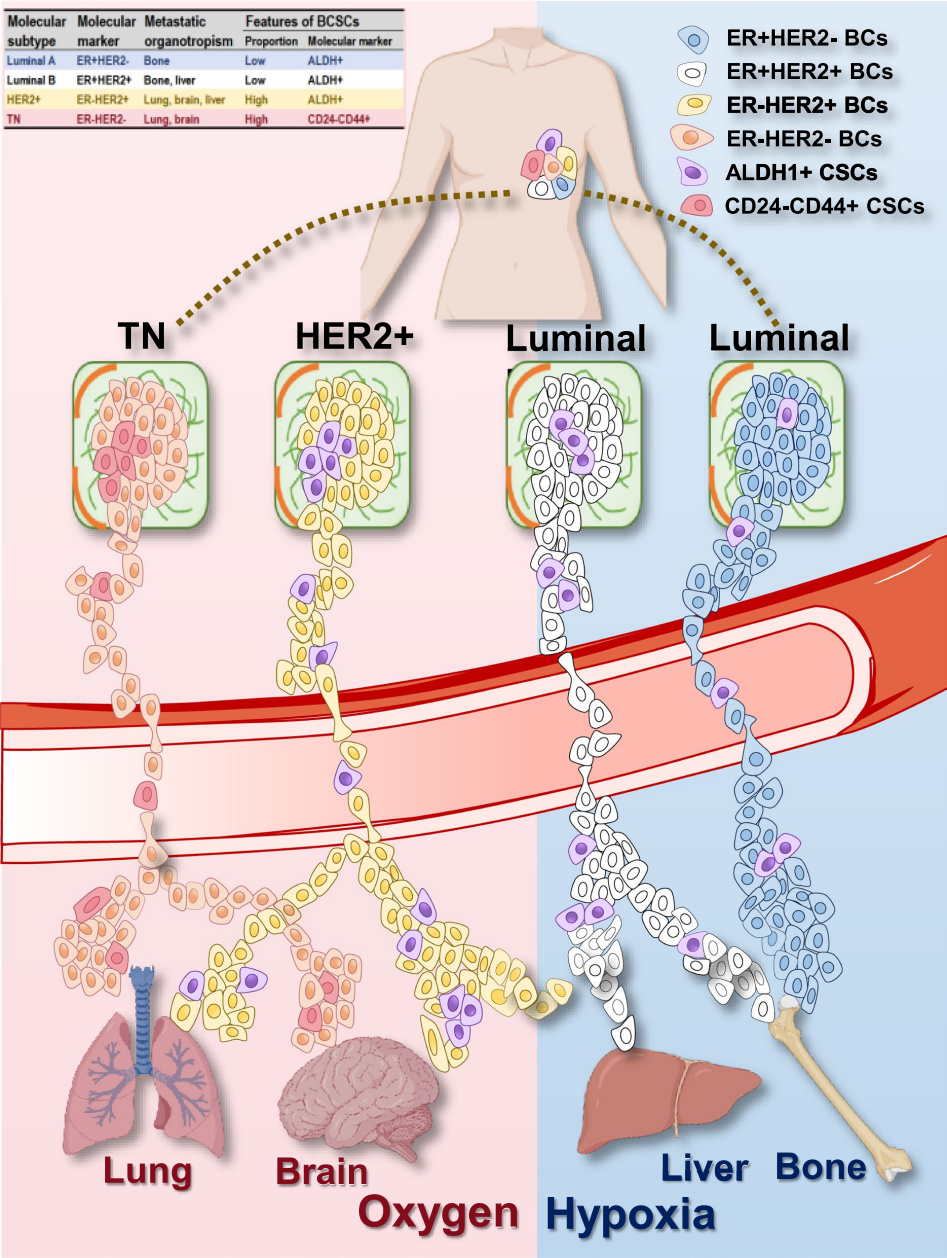
Illustrative diagram on breast cancer metastasis organotropism. Among bone, liver, brain and lung, the four organs commonly developing breast cancer metastasis, the luminal A and B subtypes are inclined to metastasize to the bone, and luminal B breast cancers may also develop liver metastasis, where both bone and liver are characteristic of hypoxia; the HER2 + and TNBC subtypes prefer metastasizing to lung and brain where oxygen supply is sufficient, yet some HER2 + tumors may also metastasize to the liver. Breast cancer stem cells (BCSCs) residing in these breast cancer subtypes differ. While luminal A, luminal B and HER2 + cancers possess BCSCs over-expressing ALDH, TNBC cells have BCSCs characteristic of CD24-CD44 + . Importantly, luminal A and luminal B cancers have low percentage of BCSCs, HER2 + and TNBC cells have high percentages of BCSCs

Bone is one of the most common sites for breast cancer metastasis, accounting for approximately 65%−75% of all metastatic cases [29]. Bone metastasis not only leads to serious skeletal complications such as pain, fracture, and hypercalcemia, but also significantly reduces the quality of life and survival time of the cancer patients [30, 31]. It has been documented that ER + breast cancers, i.e., luminal A (largely ER + HER2-) and luminal B (largely ER + HER2 +) subtypes [32–34], more easily develop bone metastasis as compared with ER- subtypes, i.e., HER2 + (ER-HER2 +) and TN (ER-HER2-) breast cancers (Fig. 2) [14]. It has been reported that 66.8% luminal A patients developed bone metastasis, whereas 38.9% TN patients having cancer cells metastasized to the bone [14]. In another study, the odds between luminal and TN breast cancers in developing bone metastasis was 8.2 [35].

Being one common site for breast cancers metastasis, 40%−50% breast tumor carriers develop liver cancer metastasis with the mortality rate being 50%−62% and the average patient survival time being 31 months [36]. Breast cancers, once metastasized to the liver, exhibit clinical symptoms such as pain in the liver region, hepatomegaly, nausea, vomiting, jaundice, and ascites. Etiology studies have indicated that breast cancers carrying HER2 positivity, i.e., luminal B and HER2 + [32–34] breast cancer carriers, are more prone to develop liver metastasis as compared with the other breast cancer subtypes (Fig. 2) [16, 37]. For example, 46.6% HER2 + breast cancer patients developed liver metastases, whereas 33% luminal A patients developed metastasis in this organ [14]; and the chance of HER2 + breast cancers in developing liver metastasis versus that of luminal A patients was 4.2/0.8 in another study [35].

Cancers, once metastasized to the brain, are lethal, leaving the percentage of patients capable of surviving for 1-year only appropriately 20% [38]. It has been estimated that 15–25% of breast cancer patients develop metastasis to the central nervous system [39]. Manifestations of brain metastasis from breast cancers include, e.g., headaches, nausea, vomiting, dizziness, seizures and increased intracranial pressure as a result of tumor formation in the brain and associated tissue compression [40]. It has been reported that ER-PR- patients, i.e., HER2 + and TN breast cancers, are more inclined for brain metastasis than luminal A/B breast cancer patients [16], with approximately 50% of HER2 + breast cancer carriers and about 1/3 of TNBC patients having gained brain metastasis, respectively, in the end (Fig. 2) [41]. It has also been reported that the rate between HER2 + tumors and the luminal A subtype in establishing brain metastasis was 1.1/0.2, and that between TN and luminal A tumors was 0.7/0.2 [35].

Lung metastasis typically does not elicit obvious symptoms until vast replacement of metastatic tumor mass occurs [42], with the primary manifestations including cough, haemoptysis, dyspnoea, malaise and weight loss [43]. Breast cancers especially ER-PR- breast cancer subtypes (i.e., HER2 + , TN subtypes) also easily develop lung metastasis, with the incidence ranging from 21 to 32% among breast cancer carriers (Fig. 2) [35]. Specifically, HER2 + breast cancer patients having received anti-HER2 targeted therapies exhibited a high risk of developing lung metastasis [44, 45], and TN breast cancers have high propensities to metastasize to the lung [42, 46]. In one study, 50.4% TN breast cancer patients developed lung metastasis whereas only 21% luminal A patients gained metastasis to the lung [14]. In another report, the ratio between HER2 + and luminal subtypes in developing lung metastasis was 3.4/1.2, and that between TN and luminal tumors was 2.5/1.2 [35].

## Critical factors defining metastasis organotropism

In the ‘seed and soil’ theory, cancer cells and the metastasized sites are referred to as ‘seeds’ and ‘soil’, respectively; while metastatic seeds can travel to any organs, they can thrive only in the congenial soil [47]. This requires organ-specific metabolic adaptation to help metastasized malignant cells overcome obstacles faced when establishing organotropism phenotypes during distant organ colonization [48], the process of which is dynamic and heterogeneous [49–51]. A variety of factors have been identified capable of, at least partially, influencing the metastasis organotropism of different breast cancer subtypes during the complicated metastatic cascade, which can be roughly categorized into cancer-intrinsic features and organ-specific microenvironment.

### Cancer-intrinsic features

Breast cancer stem cells (BCSCs) are a small cohort of breast cancer cells with the self-renewal, multipotent differentiation and tumor-initiating abilities [52]. The metastasis cascade is initiated by breaking the surrounding basement membrane and invading into the extracellular matrix (ECM), the process of which is energy-intensive [53, 54]. After departure from the primary loci, cancer cells travel in the circulation and evolve various strategies to survive in the bloodstream such as evading the immunosurveillance [55, 56]. On arrival at the sites of tropism, cancer cells may adjust to the new niche and re-initiate tumor proliferation in distant organs. Thus, disseminated cancer cells are considered to retain cancer stemness [55, 57]. Indeed, an increasing number of evidence has indicated the potential relationship between BCSCs and distant breast cancer metastasis, as well as the regulatory role of BCSCs on breast cancer metastatic organotropism. For instance, the plasticity of BCSCs allowed them to transit between the mesenchymal and epithelial states during cancer metastasis [26]. Thus, the properties of CSCs may define the metastasis organotropism from the perspective of cell-intrinsic features.

BCSCs can be grouped into three types, i.e., CD24-CD44 + BCSCs, aldehyde dehydrogenase (ALDH) + BCSCs and CD24-CD44 + ALDH + BCSCs based on the canonical CSC biomarkers used for BCSC characterization. While CD24 (a glycosylated protein linked to the cell membrane) and CD44 (a transmembrane glycoprotein interacting with various components in the ECM) play essential roles in cell adhesion and migration [58, 59], ALDH1 (a member of the aldehyde dedydrogenase family) participates in the regulation of BCSC self-renewal ability [60]. Different BCSCs are located at different positions of the invading cell collection with distinct roles. Specifically, CD24-CD44 + BCSCs are localized at the front edge of the tumor collection possessing highly invasive features, ALDH + BCSCs are located at the center of the invading population exhibiting uncontrollable proliferative properties, and CD24-CD44 + ALDH + BCSCs represent the CSC subset showing the greatest tumor-initiating capacity [61]. The stemness of these BCSCs increases from ALDH + to CD24-CD44 + to those harboring both, as only 20 BCSCs expressing both CD24-CD44 + and ALDH + effectively generated tumors whereas 100 CD24-CD44 + BCSCs and 500 ALDH + BCSCs were needed, respectively, to produce the bulk tumor cells [62, 63].

In addition, it has been estimated that up to approximately 50% of ATP was used to support the actin cytoskeleton during cancer metastasis [64, 65], leading to a positive correlation between the intracellular ATP:ADP ratio and migration potential [53]. Several cellular forces associated with bioenergetics during cancer cell migration have been identified including, e.g., integrin activation, cytoskeletal remodeling, and AMPK activation. Differentially expressed genes (DEGs) of CD24-CD44 + BCSCs and ALDH + BCSCs have been characterized, which were enriched in focal adhesion and oxidative phosphorylation, respectively [66]. These evidence have implicated the involvement of distinct molecular mechanisms in driving the mobility of different BCSCs during the onset of cancer metastasis that is consistent with the highly invasive nature of CD24-CD44 + BCSCs and the elevated glycolysis in ALDH + BCSCs [67].

Breast cancer subtypes contain different amounts and types of BCSCs, i.e. ER + (i.e., luminal A/B) cells contain relative low levels of ALDH + BCSCs [68], HER2 + cells are enriched with ALDH + BCSCs, and TNBC cells have high proportion of CD24-CD44 + BCSCs [8]. This makes TNBCs that are highly invasive exhibiting the mesenchymal-like properties, and empowers HER2 + breast cancers with the aggressive epithelial-like features [8, 69]. The exact amount of CSCs in each breast cancer subtype varies considerably among studies, possibly due to the heterogeneity of cell lines as well as the detection/quantification approaches used in each investigation. For example, ALDH1 + CSCs were reported to be 8.6%, 50%, 72.1%, 26.7% in luminal A, luminal B, HER2 + , TNBC subtypes in one study [70], and was estimated to represent approximately 52% HER2 + tumors in another study [71]. Similarly, the CD24-CD44 + CSC phenotype was manifested in 76.5% TNBCs in one study [72], and in all TNBC cells in another report [71].

### Organ-specific microenvironment

Incremental evidence has implicated that the microenvironment of the organ plays essential roles in defining the metastatic profiles of cancer cells. One reason empowering transformed cells with the invasive features is their inefficiency in getting adapted to the microenvironment of the initial organ for survival. Thus, the milieu of the secondary organ selected by cancer cells for colonization may have more favorable features for transformed cells with the corresponding tropism to survive. For instance, cancer cells initiated from an organ enriched with oxygen such as the lung may metastasize to hypoxic tissues [73], and malignant cells with a vigorous mitochondrial metabolism may leave the hypoxic liver and opt for the lung [74].

#### Bone microenvironment

Bone and bone marrow are comprised of osteocytes, osteoblasts, and osteoclasts. The bone micromilieu contains diversified cell types such as osteoblasts, osteoclasts, adipocytes, haematopoietic stem cells, and infiltrated immune cells [31]. Osteoblasts and osteoclasts play opposite roles. While osteoblasts facilitate new bone deposition to either form osteocytes or mature into lining cells, osteoclasts resorb the bone matrix. Under normal conditions, osteoblasts and osteoclasts establish a dynamic homeostasis between bone formation and decomposition [31]. Cancer cells can hijack the activities of osteoblasts and promote osteoclastogenesis to boost their proliferation and establish a vicious cycle among osteoblasts, osteoclasts, and transformed cells, since such a bone resorption process is accompanied with the release of numerous factors from the bone matrix such as cytokines, calcium, collagens, growth factors, glycoproteins, hyaluronans, proteoglycans, and proteinases [75, 76]. The inorganic part of the bone is primarily comprised of mineral hydroxyapatite crystals, and the bone matrix is enriched with bone sialoprotein, type I collagen, osteopontin, as well as a variety of growth factors and cytokines [77].

The bone microenvironment is characteristic of hypoxia [78] and acidity [79] that are interconnected. Hypoxia triggers hypoxia-inducible factor-1 (HIF-1) expression that can facilitate cancer bone metastasis (i.e., osteolytic metastasis in the case of breast cancers [31]) by promoting osteoclast generation and inhibiting osteoblast differentiation [80]. Hypoxia in the bone metastatic site can reduce pH and thus form an acidic microenvironment due to the large amount of lactic acids produced by bone metastatic cancer cells and osteoclasts [81]. Also, lactates released by breast cancer cells via glycolysis can be used by osteoclasts as the energy source [82]. Thus, breast cancers with high levels of glycolysis may be prone to bone metastasis. It has been reported that estrogen-related receptors can bind to the cis-regulatory elements of the promoters of glycolytic genes to affect cells’ energy metabolism [83]. Thus, breast caner cells over-expressing ER or PR (i.e., luminal A/B) may favor glycolysis and thus bone metastasis.

On the onset of bone resorption, the metabolism of cancer cells needs to undergo a drastic alteration from osteogenic [84, 85] to osteolytic to escape from the metastatic dormancy [4], the process of which is nourished by nutrients released from the bone matrix such as glucose, glycine, serine and glycerol [86]. The levels of several enzymes controlling de novo serine synthesis such as phosphoserine phosphatase (PSPH), phosphoglycerate dehydrogenase (PHGDH), phosphoserine aminotransferase 1 (PSAT1) and have been identified differentially elevated in breast cancer cells with high propensity for bone metastasis as compared with those without metastatic potency [87], implicating the stimulatory role of serine on osteoclast formation and osteoclastogenesis [87, 88]. On the other hand, serine is required for sustained ER signaling through regulating acetyl-CoA metabolism for histone acetylation [89]. Thus, luminal breast cancer cells harboring overt ER expression can better survive the dormancy period by satisfying the requirement on serine production for transiting cells from the osteogenic to the osteolytic state.

Another character of the bone microenvironment is high calcification deposition. Microcalcifcation in the primary site of breast cancer has been associated with matrix degradation, inflammation, and cell proliferation and migration [90]. Breast microcalcifcation, being subgrouped into type I and II that are composed of calcium oxalate crystals and hyaluronan respectively [90], has been commonly used for breast cancer screening and diagnosis. Thus, breast cancer cells capable of increasing the intracellular concentration of calcium ion through phagocytosis and/or degrading intracellular crystals may better survive the bone microenvironment and colonize in the bone [91]. Importantly, estrogen can regulate intestinal calcium absorption via differential roles of ERα and ERβ on duodenal epithelial cellular plasma membrane calcium ATPase (PMCA) and transient receptor potential cation channel subfamily V member 6 (TRPV6) [92]. The effects of estrogenic agonists on stimulating acute calcium signaling have been previously reviewed in the cardiovascular system [93]. In addition, breast cancer cells preferring bone metastasis can sense the extracellular calcium concentration via calcium-sensitive receptors for promoted migration [94–96]. These have, collectively, implicated and explained the propensity of luminal breast cancers towards bone metastasis.

#### Liver microenvironment

The liver microenvironment is primarily composed of hepatic stellate cells (HSCs), hepatocytes, sinusoidal endothelial cells and various types of immune cells including metastasis-associated macrophages [97]. In particular, metastasis-associated macrophages can secrete granulin to activate HSCs that, once activated, sustain tumor growth by generating a fibrotic and immune-permissive milieu, and facilitate ECM degradation by secreting a panel of growth factors and cytokines [98–100].

One critical characteristic of the liver microenvironment is hypoxia [101].This makes cancer cells translocated to the liver exhibiting a similar hypoxic glycolytic metabolic profile with the local hepatic cells, i.e., elevated glycolytic activity and reduced mitochondrial metabolism. A study comparing the metabolisms of breast cancer cells metastasized to different organs reported that transformed cells migrated to the liver displayed enhanced glycolysis, among other features, which can be attributed to the increased expression of HIF-1α [101]. Besides, oxidation phosphorylation and glutamine metabolism are weaker in cells colonized in the liver, further facilitating the adaptation of cancer cells to the hypoxic milieu in the liver [101]. Thus, the highly hypoxic liver microenvironment forces cells capable of surviving the liver milieu to evolve the feature of favoring anaerobic glycolysis, i.e., the Warburg effect. On the other hand, it is known that ALDH1A3 promoted pancreatic cancer metastasis by enhancing cellular glycolysis [102], implicating that breast cancer cells harboring ALDH1 over-expression (i.e., the HER2 + subtype) are easier to adapt to the microenvironment of the liver.

Another feature of the liver microenvironment is the production of ketone bodies that cannot be consumed by normal adult hepatocytes [103]. Ketone bodies can be catalyzed to acetyl-CoA through ketolysis for energy production under glucose shortage [104]. It has been underscored that simultaneously targeting fatty acid oxidation and HER2 is feasible for treating HER2 + breast cancers or, in other words, blocking ketolysis can sensitize HER2 + tumors to the corresponding targeted therapies [105]. This implicates the metabolic plasticity of HER2 + breast cancer cells that can be fueled by both glucose and ketone bodies, and also explains the organotropism of the HER2 + subtype towards liver.

The third unique feature of the liver microenvironment is its high accessibility due to its dual blood supply system. In particular, liver receives the blood supply from both hepatic arteries and hepatic portal vein, and thus has a much lower sinusoid blood pressure gradient [106, 107].This unique architecture of liver makes traveling cancer cells easier to attach to the sinusoidal endothelium for seeding. It has been observed that HER2 status is the only breast cancer receptor correlated with the presence of circulating tumor cells [108], suggesting that HER2 + breast tumor cells are the easiest, among other subtypes, to gain the migration ability. Thus, it is no wonder that HER2 + breast cancers prefer metastasizing to the liver given their abundance in the blood stream.

#### Brain microenvironment

The brain parenchyma is composed largely of neurons and glial cells (i.e., astrocytes, microglia, oligodendrocytes) [109]. Among these cells, the roles of astrocytes during brain metastasis are the best-understood. For instance, astrocytes secrete growth factors and cytokines to support neurons, the ability of which can be hijacked by TNBC (MDAMB231) cells to favor metastasis [110, 111]. Specifically, presynaptic neurons release glutamates that are rapidly absorbed by astrocytes and postsynaptic neurons during glutaminergic synapses [112], and metastatic TNBC cells (MDAMB231) can use glutamates released by neuronal cells to activate N-methyl-D-aspartate (NMDA) receptors and form pseudo-tripartite synapses with glutamatergic neuron cells for promoted brain colonization [113]. Oligodendrocytescan can interact with neurons to drive myelin plasticity and participate in the crosstalk between neurons and tumor cells for promoted tumor cell survival and colonization [114]. Microglia, also known as macrophages residing in the brain, facilitate breast cancer cell colonization in the brain via serving as active transporters and guiding rails in a Wnt-dependent manner [115].

One critical characteristic of the brain microenvironment is high oxygen supply and oxidative stress as a result of a high oxygen consumption to satisfy its high demand on energy turnover [116]. Thus, breast cancer cells colonized in the brain displayed a high plasticity regarding pathways used for energy supply that include, e.g., glycolysis, oxidative phosphorylation, pentose phosphate pathway, gluconeogenesis, branched chain amino acid oxidation [117], and acetate metabolism [118]. In consistent with this, cancer cells capable of surviving in the brain utilize fuels from both glucose and/or ketone bodies [116], with glucose being the primary source for generating the energy and nutrients including lactic acids and nonessential amino acids such as alanines, gamma aminobutyric acid (GABA), glutamic acids, glutamines and glycines [119]. Evidence supporting the essential role of lipid metabolism in brain metastasis include, e.g., the aid of FABP7 (a member of the FABP family actively participating in fatty acid metabolism) in the glycolysis and lipid droplet storage process of HER2 + breast cancer cells (BT474) for surviving the brain microenvironment [120]. Besides satisfying the requirement of a constant high energy supply, cancer cells capable of colonizing in the brain exhibited improved glutathione (GSH) system to respond to the high oxidative stress [121]. For instance, GABA catabolism has been reported to be utilized by HER2 + (SKBR3) and TNBC (MDAMB231) cells metastasized to the brain for enhanced nicotinamide adenine dinucleotide (NADH) generation [122].

One unique feature of the brain is its blood–brain barrier (BBB) that is a non-fenestrated endothelium stitched together by tight junctions and supported by astrocytes, pericytes and a basement membrane [123]. The BBB protected the brain from being invaded by cancer cells. Thus, malignant cells capable of metastasizing to the brain need to evolve abilities of transmigrating through the BBB, with several strategies destroying the BBB being identified. For instance, TNBC (MDAMB231) cells produced cathepsin S to proteolyze the junctional adhesion molecule JAM-B [124], and secreted miRNA-181c-enriched extracellular vesicles to down-regulate phosphorylated cofilin for interrupted actin dynamics [125], which ultimately led to BBB breakdown.

#### Lung microenvironment

The lung microenvironment is composed of varied types of cells including, e.g., alveolar epithelial cells that can be subgrouped into type I and type II, type II alveolar epithelial cells, endothelial cells, fibroblasts, and various kinds of immune cells including alveolar macrophages [126]. Resident cells in the lung can establish a pro-metastatic niche by, e.g., secreting abundant chemokines such as CXCL12 and CCL21 to attract breast cancer cells over-expressing their receptors CXCR4 [127] and CCR7 [128] to the lung [129]. Alveolar macrophages, of note, facilitate lung metastasis by suppressing T cell responses and producing the pro-inflammatory mediator leukotriene B4 (LTB4) that is a potent lipid chemoattractant driving neutrophil swarming and leukocyte migration [130, 131].

One critical characteristic of the lung microenvironment is the oxidative stress. In contrast to the micro milieus of the bone and liver, lung is the respiratory organ responsible for oxygen inhale and carbon dioxide exhale and thus contains high oxygen levels. Therefore, transformed cells need to overcome the oxidative stress before colonizing in the lung [132–134]. Various strategies have been evolved by metastatic cancer cells to adapt to the lung microenvironment. For instance, TNBC (MDAMB231) cells can counteract electron leakage and ROS generation by up-regulating peroxisome proliferator-activated receptor gamma coactivator 1 alpha (PGC-1α) for improved mitochondrial biogenesis [132], and elevating the expression of antioxidant proteins such as peroxiredoxin 2 (PRDX2) [135]. Alternatively, reprogramming the metabolism to favor abundant oxygen supply represents another strategy of cancer cells to get immune to the oxidative stress. For example, increased activity of pyruvate carboxylase (PC), an enzyme catalyzing pyruvate to produce oxaloacetate for gluconeogenesis and TCA cycle replenishment, has been identified for lung metastatic TNBC-like (i.e., 4T1) cells towards increased glycolysis and oxygen consumption [136].

Another feature of the lung microenvironment is its tightly connected capillary endothelial cell wall. For effective oxygen inhale and exhale, the lung has developed an abundant capillary network. Traversing such a capillary barrier is required for cancer cells to colonize in the lung [126]. One strategy evolved by transformed cells (3LL-LLC, a mouse Lewis lung carcinoma cell line) is to induce increased expression of matrix metalloprotein 9 (MMP9, a protein responsible for ECM breakdown) in lung endothelial cells and macrophages via the VEGFR-1/Flt-1 tyrosine kinase (TK) axis [137].

The third characteristic property of the lung microenvironment is its metastasis-suppressive niches capable of inhibiting cancer cell growth [126]. In particular, a perivascular niche was identified capable of inducing sustained quiescence among breast cancer cells metastasized to the lung, bone marrow and brain [138]. The inhibitory effect of this metastasis-suppressive niche is attributable to endothelial-derived thrombosondin-1 (TSP1) [138]. In addition, CX3C-chemokine receptor 1 (CX3CR1) + monocytes were recruited by the ligand of CX3CR1 derived from lung endothelial cells (i.e., CX3CL1) to activate natural killer (NK) cells for prevented lung metastasis [139]. Also, bone morphogenetic proteins (BMPs) originated from lung fibroblasts had an inhibitory effect on the self-renewal abilities of CSCs [140, 141]. Tumor cells have evolved various approaches to overcome such a growth-suppressive environment for survival. One strategy widely adopted by tumor cells is to augment the cancer stemness through secreting relevant proteins. For instance, TNBC (MDAMB231) cells have been shown capable of producing polypeptide N-acetyl-galactosaminyltransferase 14 (GALNT14) that augments the self-renewal properties of breast cancer cells via exploiting macrophage-derived fibroblast growth factors (FGFs) and inducing macrophage infiltration [142]; and TNBC (MDAMB231) cells metastasized to the lung could secrete tenascin C (TNC), an protein residing in the stem cell niche and sites of epithelial-mesenchymal interactions [143, 144], to amplify stem cell-related signalings such as the Wnt and Notch pathways [145]. Another strategy used by TNBC (MDAMB231) cells is to secrete extracellular vesicles and transform the residing cells in the milieu to foster a metastatic-promotive niche. For example, TNBC-derived exosomes (using MDAMB231 as the modeling tumor cell line) expressing integrins α6β4 and α6β1 promoted the expression of the pro-inflammatory gene *S100A4* and activated the oncogenic protein Src in resident cells [146]; and chemotherapy-induced extracellular vesicles secreted by TNBC cells (i.e., MDAMB231, 4T1) were enriched with annexin A6 (ANXA6), which is a Ca^2+^-dependent protein capable of activating NFƙB-dependent endothelial cells and expanding Ly6C + CCR2 + monocytes [147].

### Organ redox milieu and cancer metabolic profile define metastasis organotropism

From the perspective of the ‘soil’ or organs frequently being metastasized by breast cancer cells (i.e., bone, liver, brain, lung), bone and liver are relatively hypoxic, brain and lung are enriched with oxygen. Accordingly, breast cancer cells favoring bone and liver metastasis are largely luminal A and B subtypes, and those preferring brain and lung are primarily HER2 + and TNBC cells. In particular, the oxidative status increases from bone, to liver, to brain, and to lung, which gradually corresponds to the organotropism of luminal A, luminal B, HER2 + and TNBC cells (Fig. 2).

From the perspective of the ‘seed’ or breast cancer cells, the cancer stemness and metabolic plasticity of these cells increase from luminal A and luminal B, to HER2 + and TNBC cells, with a giant discrepancy being occurred between the luminal (luminal A/B) and non-luminal (HER2 + and TNBC) subtypes. In other words, while luminal A and B cells can solely resort to the Warburg effect or anaerobic glycolysis to fuel their energy production, HER2 + and TNBC cells can effectively adapt to the oxidative stress imposed by high oxygen exposure and switch to aerobic glycolysis. Indeed, CSCs are known to favor glycolysis instead of oxidative phosphorylation for energy production [148], and aerobic glycolysis is metabolically more efficient than anaerobic glycolysis in producing ATP [149]. On the other hand, as CSCs have a more robust anti-oxidation machinery than the bulk tumor cells that render them easier to survive the oxidative stress, breast cancer cells possessing higher percentages of BCSCs are more easily to colonize in oxygen-enriched organs such as the brain and lung. The type of BCSCs harbored by breast cancer cells also plays a role in the organotropism of transformed cells. By comparing HER2 + and TNBC cells that are both enriched with BCSCs, HER2 + cancer cells possess a high proportion of ALDH + BCSCs and display more epithelial-like features, and TNBC cells harbor a high percentage of CD24-CD44 + BCSCs and manifest more mesenchymal-like characteristics [8, 66]. Accordingly, besides brain and lung that can be colonized by both HER2 + and TNBC cells, HER2 + cancers can also easily metastasize to the liver whereas TNBC cells do not [8]. Recall the vital role of epithelial-mesenchymal transition (EMT) during the metastatic process [150, 151], these evidence suggest that ALDH + BCSCs are more tolerant to hypoxia than CD24-CD44 + BCSCs for sustained metabolism or, in other words, ALDH + BCSCs have a less tendency than CD24-CD44 + BCSCs in getting metastasis. Such a difference is predisposed by the markers characterizing these BCSCs. That is, ALDH + BCSCs can rely on oxidative phosphorylation for sufficient energy supply, since ALDH is an enzyme involved in glycolysis [66]; and CD44 can aid in tumor migration by participating in the crosstalk between cancer cells and their ECM, given that it is a multi-functional surface adhesion receptor [59].

**Fig. 3:**
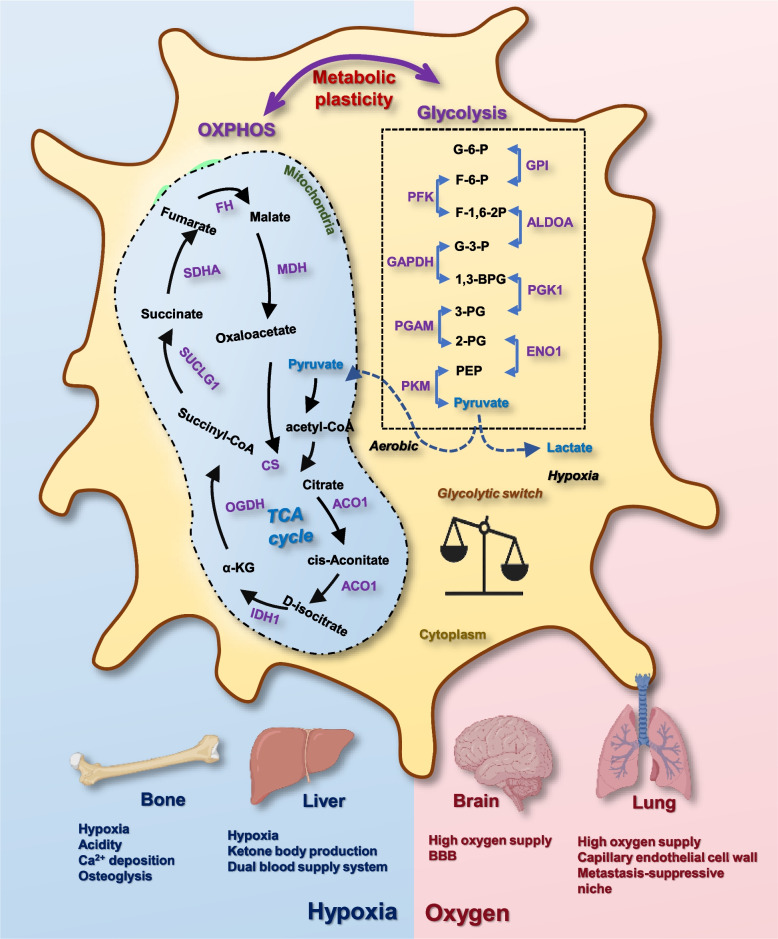
Factors determining cancer cell metastasis organotropism. The metabolic profile of cancer cells and the redox status of the organ microenvironment dynamically interact during the metastasis cascade to collectively determine cancer metastasis organotropism. Specifically, cancer cells possessing high percentages of CSCs have high metabolic plasticities, since CSCs are able to produce cancer cells relying on different metabolic programs for energy production. Thus, breast cancer subtypes containing high levels of BCSCs (i.e., HER2 + , TNBC) have high metabolic plasticities and can metastasize to organs with sufficient oxygen supply (brain, lung); and subtypes harboring low levels of BCSCs (i.e., luminal A, luminal B) are more inclined to develop bone and liver metastasis that are characteristic of hypoxia. In addition to the redox status of the organ microenvironment, other features of the organ milieu may also affect cancer metastasis organotropism. Besides being hypoxic, bone is featured by acidity that is accompanied with hypoxia, osteolysis that provides nutrients for cancer cell survival, and Ca^2+^ deposition that can be regulated by estrogen; liver is characteristic of ketone body production that supply nutrients for cancer cell survival, and dual blood supply system that favors cancer cells with high abundance in the blood (i.e., HER2 +). Besides having sufficient oxygen supply that may impose the oxidative stress, brain and lung have characteristic barriers that allow only cancer cells that have evolved the corresponding strategies to colonize, i.e., the unique blood–brain barrier (BBB) in the brain and the capillary endothelial cell wall and metastasis-suppressive niche in the lung

Therefore, the organotropism of cancer cells is collectively dictated by the redox milieu of the organ and the metabolic profile of metastasized cancer cells (Fig. 3). In other words, cancer cell organotropism is a determination of transformed cells possessing a certain level of metabolic plasticity on whether or not to be ready for colonization in response to the redox levels of the organ microenvironment; and a selection of cancer cells harboring the metabolic properties feasible to be adapted to the redox status of the organ. Indeed, altered redox regulation has been viewed as an essential force driving cancer cell metastasis [152]. Provided with the inefficiency of the migration process where the vast majority of the transformed cells are fated to death, cancer cells capable of traveling through diverse redox environments must possess a highly versatile metabolic profile empowering them with the immunity to the oxidative stress. Cancer cells with the metastatic potential must have higher percentages of CSCs than the bulk tumor cells given the pluripotency of this small cell cohort that can generate tumor cells with diverse metabolic patterns to meet the requirement of a new environment. It is thus no wonder that cancer cells with metastatic potential possess higher cancer stemness, and cancer cells with the organotropism for highly oxidative organs such as brain and lung are more stem-like and more malignant as compared with their peers containing more bulk tumor cells.

Insights gained from breast cancer organotropism apply to other types of cancers. That is, the higher metabolic plasticity that cancer cells possess the higher stemness these transformed cells harbor. For example, the metabolic signature of hepatocellular carcinoma cells with stable knockdown of *EphA2* and *Acly* (two genes with demonstrated effects in promoting the aggressiveness and self-renewal ability of cancer cells) was significantly enriched with fatty acid, cholesterol and TCA metabolite profiles and accordingly, Ephrin-A3/EphA2 signaling was identified as an important molecular axis in regulating the metabolic plasticity of hepatocellular carcinoma cells to enhance cancer stemness [153]. As another example, β-hydroxybutyrate significantly enhanced the self-renewal and migration potential of colon cancer cells, the process of which involved decreased extracellular acidification rate (ECAR) and expression of genes related to glycolysis, as well as increased oxygen consumption rate (OCR) and expression of genes associated with mitochondrial biogenesis and cancer stemness [154]. Similarly, metabolic plasticity has been considered as one primary source driving the stemness of pancreatic cancer cells, with the loss of mitochondrial ISGylation (a post-translational modification process regulated by interferon-stimulated gene 15) being a concomitant event occurring along with reduced oxidative phosphorylation (OXPHOS) and decreased amount of cancer stem cells [155]. Consistent with this, another study reported that bioenergetic modulators such as 2-deoxyglucose, dichloroacetate and phenformin decreased the CSC percentage of pancreatic ductal adenocarcinoma cells by switching its metabolic mode from glycolysis to glutamine metabolism [156].

## Cancer cell vulnerability during metastasis

### Metabolic plasticity and cancer stemness

**Fig. 4:**
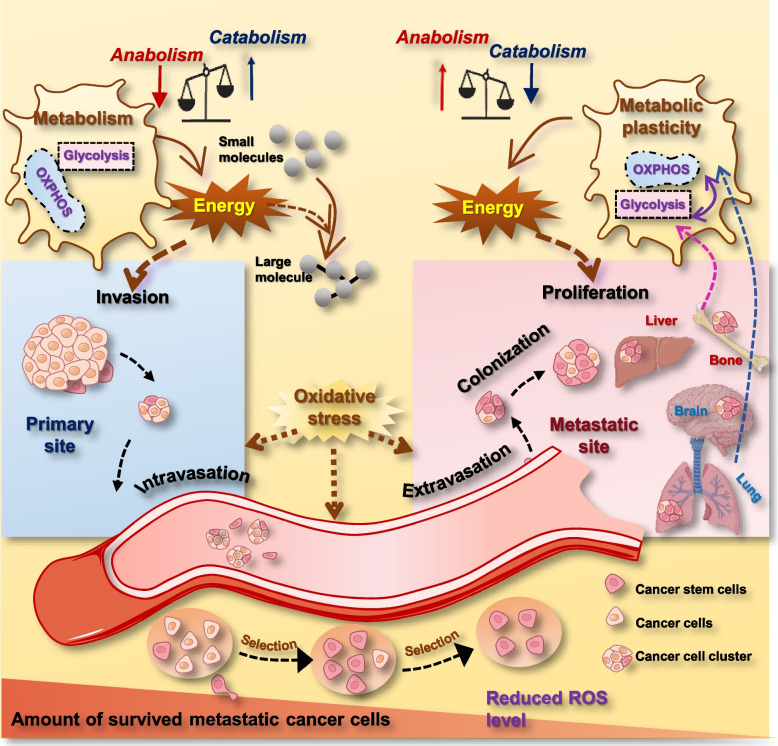
Metastatic cells are subjected to oxidative stress selection and are vulnerable to pro-oxidant therapeutics. During the metastasis cascade (i.e., invastion, intravasation, circulation, extravasation, colonization), cancer cells are subjected to the selection of the oxidative stress, resulting in declined number of cancer cells and enrichment of cancer stem cells. These cancer stem cells are featured with reduced level of ROS and enhanced metabolic plasticity. Under the selection power of oxidative stress, cells with reduced intracellular ROS generation and reduced basal redox level easily survive. When arriving at the secondary metastatic site, cells with high metabolic plasticity can easily get adapted to the microenvironment of the metastatic site, i.e., cells rely more on glycolysis when the microenvironment is hypoxic, and rely more on oxidative phosphorylation when the microenvironment is oxidative. In the case of breast cancers, TN and HER2 + breast cancer cells that have high metabolic plasticity can easily get adapted to the oxidative microenvironment of brain and lung and thus rely more on OXPHOS for energy production after colonization, and luminal A/B breast cancer cells more easily develop liver and bone metastasis and favor glycolysis for energy production once colonized in the secondary lesions. Also worth mentioning is that successful metastasis often occurs in the form of cell clusters and is accompanied with suppressed anabolism in the beginning that regains the activity after colonization. In the beginning of the metastasis cascade, cells need lots of energy to accomplish invasion, intravasation, circulation and extravasation, leading to reduced amount of energy used for anabolism. Once metastatic cancer cells have colonized to the new environment and grown beyond a few millimeters in diameter, they regain the ability of rapid growth as a result of fully activated anabolic processes

The high metabolic plasticity and oxidative damage resistance of CSCs can be attributed to the evolutionary selection during metastasis that is fated to happen when the primary tumor site could not meet the nutritional requirement of the ever-growing tumor body (Fig. 4). Oxidative stress functions as the selection power to limit the amount the cancer cells during metastasis [134]. It has been reported that antioxidants increased the number of circulating tumor cells in the blood and metastatic tumor sites [134, 157–159], and metastatic cancer cells undergoing metabolic alterations have been perceived with reduced levels of ROS generation [121, 160–164].

### Oxidative stress as a selection pressure

The selection force imposed by the oxidative stress is likely to happen via reducing the ability of metastatic cells to fully reactivate the anabolic pathways required for tumor growth (Fig. 4). For instance, nicotinamide adenine dinucleotide phosphate (NADPH) and its reducing equivalents are needed for cancer cells to combat with the oxidative stress; and this leads to inhibited acetyl-CoA carboxylase and thus decreased fatty acid synthesis (an anabolic process) for reduced NADPH consumption [165]. Nonetheless, once metastatic tumor cells have adapted to the oxidative stress and grown beyond a few millimeters in diameter (i.e., having survived the quiescence stage), they regain the ability of rapid growth that requires a broad activation of the anabolic processes.

The proposed insights here underpin the imprinting role of cell metabolism in empowering cells with discrepant manifestations, some of which may ultimately drive carcinogenesis. Thus, gaining mechanistic insights into the dynamic process of oxidative stress in stratifying and editing the metabolic plasticity of transformed cells may help identify critical clues for the establishment of onco-therapeutics with improved efficacy and safety.

## Pro-oxidative approaches in cancer control

### Existing pro-oxidative onco-therapeutics

Antioxidant supplementation has been proposed to provide health benefits via reducing ROS levels [166]. Despite consecutive clinical efforts examining the efficacy of antioxidants in halting carcinogenesis, no supportive evidence has been reported regarding the hypothesized roles of antioxidants in reducing cancer incidence or cancer-related deaths [167]. Instead, both pre-clinical and clinical data have suggested that antioxidants tend to promote cancer development and increase cancer-related deaths [168–170]. This leaves us the therapeutic opportunity to target cancer cells via imposing a high selection force during cell metastasis, especially before they have successfully colonized and adapted to the redox environment of the targeted sites. In principle, when the selection pressure is sufficiently high, cancer cells fail to metastasize that are eventually restricted to their primary site and become benign.

**Fig. 5:**
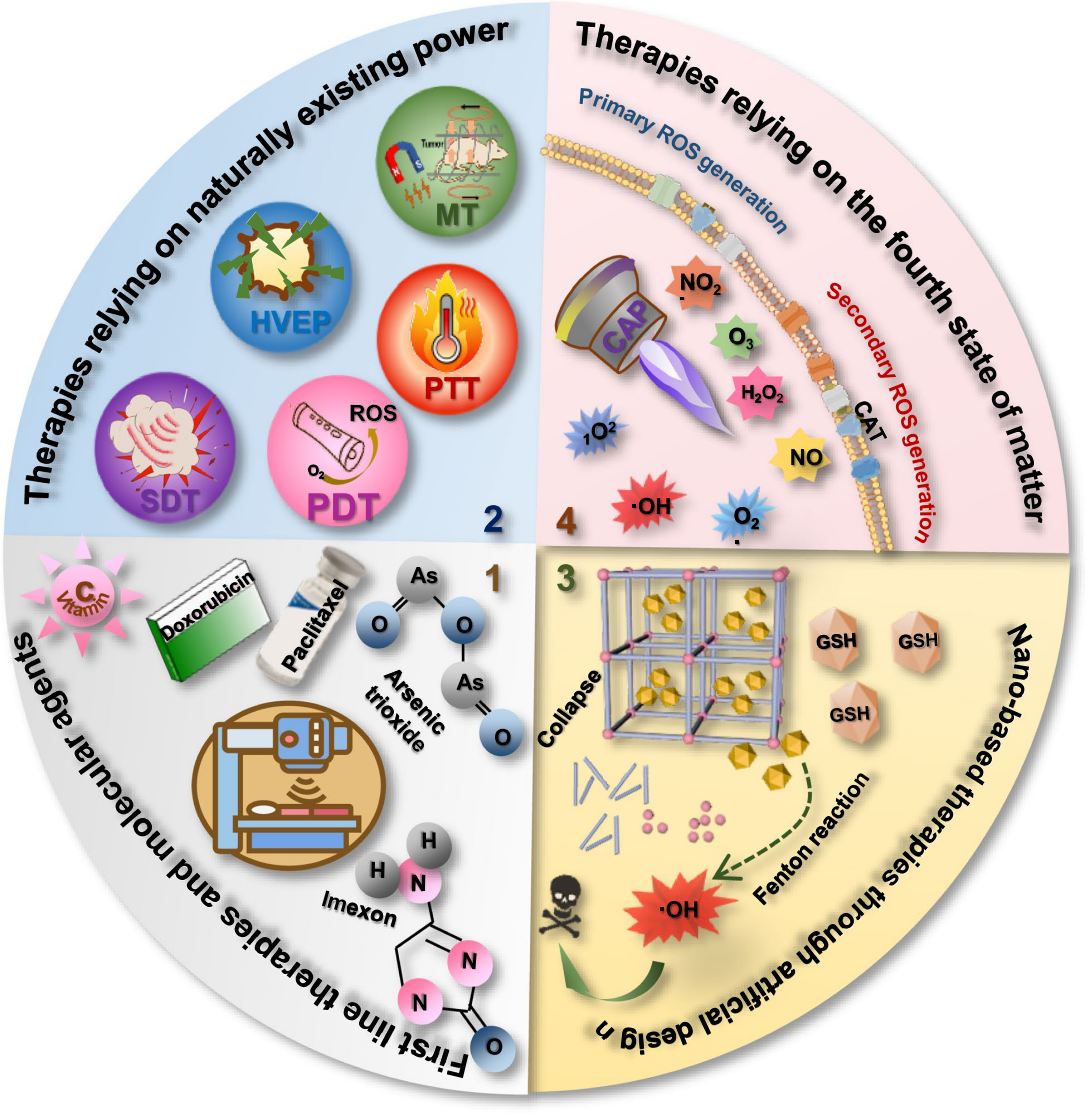
Pro-oxidative approaches for treating cancers. Current pro-oxidative approaches for cancer treatment can be categorized into four types, i.e., first line therapies and molecular agents, therapies relying on naturally existing power, nano-based therapies through artificial design, and therapies relying on the fourth state of matter. Example therapeutics of the first category include chemotherapies such as paclitaxel, doxorubicin, imexon, arsenic trioxide, radiotherapies, and high dose of vitamin C. Therapeutics of the second category can be further divided into magnetic therapy (MT), high-voltage electrical pulse (HVEP), photodynamic therapy (PDT), photothermal therapy (PTT), and sonodynamic therapy (SDT), which take advantages of the magnetic field, electrical field, light, heat and sound, respectively. One example of the third category is GSH-responsive metal–organic-framework (MOF) that generates ROS via Fenton reaction. Cold atmospheric plasma (CAP) belongs to the fourth state of matter and the fourth category of pro-oxidative therapeutics. CAP is composed of a cocktail of reactive oxygen and nitrogen species such as OH•, O_2_•, _1_O^2^, H_2_O_2_, O_3_, NO, NO_2_^−^. These species take actions via interacting with the cell surface to generate primary and secondary ROS. For example, long-lived species H_2_O_2_ and NO_2_^−^ from CAP can interact to generate ONOO^−^. In the vicinity to membrane-associated proton pumps, ONOO^−^ is protonated to ONOOH and decomposed into ·NO_2_ and ·OH. ·OH reacts with H_2_O_2_ to form HO_2_·. The subsequent generation of O_2_NOOH and O_2_NOO^−^ allows for the generation of primary ^1^O_2_. Primary ^1^O_2_ causes local inactivation of CAT. Surviving H_2_O_2_ and ONOO^−^ at the site of inactivated CAT further generate secondary ^1^O_2_. Secondary ^1^O_2_ can, in turn, inactivate CAT to trigger ^1^O_2_ auto-amplification and promote secondary ^1^O_2_ generation

It has been reported that pro-oxidant therapies can arrest cancer progression via exacerbating the oxidative stress in cancer cells and/or blocking the metabolic adaptation process to confer oxidative stress sensitivity [171]. Many existing onco-therapeutic strategies can be considered fell into this kind, including the primary options for cancer treatment, i.e., chemotherapy and radiotherapy [172–178] (Fig. 5). Specifically, chemo-therapies can all impose oxidative stress to cancer cells [175–177]. For example, paclitaxel (a herbal-derived agent used for treating a plethora of cancers such as breast cancer, ovarian cancer, cervical carcinoma, endometrial cancer, advanced prostate cancer, bladder cancer, and non-small cell lung carcinoma [179]) can activate phosphatase and tensin homolog deleted on chromosome ten (Pten) and thus suppress the phosphoinositide 3-kinase (PI3K) axis via producing overt amount of ROS [179]; doxorubicin, an anthracycline type of chemotherapeutic agent canonically used for treating cancers such as breast carcinoma, lymphoma and sarcoma, can produce excessive ROS to induce lipid peroxidation as well as damage DNA and protein [180]; imexon, a small molecule capable of depleting GSH and increasing ROS levels via binding to thiols, has been examined for activity against non-Hodgkin lymphoma [181]; arsenic trioxide, capable of causing electron leakage and superoxide anion radical (O^2•−^) generation by impairing the function of the electron transport chain, has been used for treating acute promyelocytic leukemia [182]. Radio-therapies are known capable of generating O^2•−^, hydroxyl radicals (OH•) and hydrogen peroxide (H_2_O_2_) [178, 183] to take on their cytotoxic effect [184], and inducing endogenous ROS production in mitochondria to trigger intracellular redox imbalance [185]. However, these commonly seen pro-oxidative onco-therapeutic approaches are characteristic of unavoidable severe side effects and drug resistance [186], rendering the development of innovative therapies capable of alleviating these disadvantages an ever-lasting focus in the field of oncology.

Some antioxidants such as vitamin C, once given at a dose far exceeding that is recommended for daily intake, turn to be pro-oxidative. Vitamin C (also named ascorbate) is canonically considered as an antioxidant supplement for the benefits of health; yet it becomes pro-oxidative once infused intravenously at a high dose [187]. This is because that superphysiologic levels of vitamin C can be uptaken by cancer cells in its fully oxidized form, dehydroascorbate, via the glucose transporter 1 (GLUT1) expressed on the membrane of cancer cells; after entering the cancer cells, dehydroascorbate is reduced back to ascorbate, the process of which consumes the reducing equivalents and induces cellular oxidative stress [188, 189]. Given the little toxicity reported, high dose vitamin C seems to be promising in treating cancers as an adjuvant therapy. However, its additive anticancer impact still remains debatable that requires rigorous clinical validations [190]. In this context, increasing attention has been paid to naturally existing matters such as magnetic field, electrical field, light, and ultrasound in treating cancers that are, in principle, of high efficiency and low low cost.

Magnetic fields can be categorized into static magnetic field (also named constant magnetic field) or dynamic magnetic field (Fig. 5). Static magnetic fields can be produced by permanent magnets or solenoidal coils with unidirectional currents, and be classified into weak (< 1 mT), medium (1 mT to 1 T), strong (1 to 5 T), and ultra-strong (> 5 T) magnetic fields [191]. Dynamic magnetic fields vary with time and can be grouped as an alternating magnetic field, pulsed magnetic field, pulsating magnetic field, and geomagnetic field. While alternating magnetic fields can be produced by an electromagnetic coil with a current of a certain frequency or through a magnet with regular motion, pulsed magnetic field, pulsating magnetic field and geomagnetic field can be generated by an electromagnetic coil with a pulsed current, alternating current power supply, and Earth and ionosphere, respectively [191]. Magnetic fields at frequencies below 100 kHz could non-invasively induce the death of cancer cells without causing the thermal effect [186, 192]. Thus, static magnetic fields and low-frequency magnetic fields (frequency < 300 Hz) have been shown promising for treating a variety of cancers pre- or clinically including, e.g., breast cancer [193], colon carcinoma [194], Lewis lung carcinoma [195], gastric cancer [196], Ehrlich ascites carcinoma [197], and fibrosarcoma [198]. Magnetic therapies take actions via promoting the generation of ROS [199, 200]. For instance, treating human prostate cancer cells with a 60 Hz sinusoidal magnetic field for 48 h resulted in ROS accumulation that ultimately led to the apoptosis of transformed cells [201]. Despite the increasing evidence supporting the inhibitory effect of magnetic fields on tumor progression, contradictory reports exist. For example, long-term exposure of 7,12-dimethylbenz(a)anthracene (DMBA)-treated female rats to an alternating magnetic field of low flux density promoted the growth and increased the incidence of mammary tumors [202]; and there was some evidence for elevated risk of developing leukemia among children aged above 5 years old living in the proximity to transformer stations [203–205]. These underpin the complicated mechanisms driving the impact of magnetic fields on cancer cells that are far from being completely understood, and such uncertainties regarding the treatment outcome have substantially hindered the clinical translation and wide application of magnetic therapies.

High-voltage electrical pulse (HVEP), taking advantages of concentrated energy induced by electric pulses, has been shown promising in ablating cancers especially those difficult-to-treat solid tumors (Fig. 5). By varying the electrical field strength and treatment duration, electroporation can be either reversible or irreversible. In general, the energy used for generating electrical pulses of irreversible HVEP is much more intensive than that is required for producing reversible HVEP. That is, at least an amplitude up to 3000 V and 80–100 pulses are required for irreversible HVEP to be effective in treating cancers, and an amplitude of 100–1000 V and 8 square wave pulses of 100 μsec are typically used in generating reversible HVEP [206]. Accordingly, the effect of irreversible electroporation is lethal, and that of reversible electroporation is transient that is restricted to the impact on cell membrane permeability. Increased ROS cellular levels has been associated with HVEP. For instance, a statistically significant ROS increase was observed 3 h after electric pulse exposure in glioblastoma cells cultivated using the standard culture condition, and was shown immediately post-exposure in glioblastoma cells cultivated as neurospheres [207]. Lots of clinical evidence has suggested the feasibility of using irreversible HVEP in ablating hepatoma [208–211], kidney carcinoma [212, 213], prostate cancer [214–216] and pancreatic cancer [217–224]; and of using reversible HVEP in treating large size cutaneous metastases of all cancer types [225]. However, HVEP therapies are lack of sufficient targeting ability and may impair the functionality of certain organs receiving the treatment. For example, irreversible electroporation may cause prostate cancer patients loosing up to 41% potency post-treatment [226].

Photodynamic therapy (PDT) is known to rely on the generation of cytotoxic ROS on the ignite of a specific light wavelength in killing transformed cells (Fig. 5). In principle, the photosensitizer administrated to the tumor site can react directly with nucleic acid, protein, or unsaturated lipid to generate ROS such as O^2•−^, OH•, or hydrogen peroxide (H_2_O_2_) in the presence of oxygen (O_2_) through proton or electron transfer (namely the type I reaction); the excited photosensitizer can react with molecular O_2_ to form singlet oxygen (_1_O^2^) via energy transfer (called type II reaction), where _1_O^2^ is a leading ROS taking actions in PDT [227, 228]. The anticancer efficiency of PDT largely depends on the properties of photosensitizer that is expected to preferentially accumulate in the tumor tissues. However, as the tumor-targeting feature of a photosensitizer is actualized largely through a passive rout, i.e., via the permeability retention (EPR) effects in tortuous blood vessels and leaky vasculature, one major disadvantage of PDT is the retention of photosensitizers in cells that can last for several weeks [229]. During this period, the skin and eyes can become extremely sensitive to light and quickly become swollen, sunburned, and blistered [230]. Besides, the ROS generation of photosensitizers varies in vivo, rendering it difficult to determine the light dose sufficient to induce PDT in a particular tumor loci without causing photobleaching reactions. Another obstacle faced by PDT is the limited penetration in-depth of light, i.e., approximately 10 mm, restricting its use for treating visceral tumors.

Ultrasound-based sonodynamic therapy (SDT), using sonosensitizers and ultrasound in cancer treatment, also relies on ROS generation in killing cancer cells (Fig. 5). It differs from PDT in having a deeper tissue penetration ability, i.e., 70–100 mm, and thus has become an attractive option for treating solid malignant tumors [231] and be applied in treating a broader range of cancer types including, e.g., liver cancer [232], breast cancer [233, 234], glioblastoma [235], colon cancer [236], and pancreatic cancer [237]. Similar to PDT, the efficacy and safety of SDT are primarily determined by the properties of sonosensitizers, and appropriate ultrasonic dose setting is vital in reaching the desirable therapeutic outcome [238]. Thus, sonosensitizer screening and state-of-the-art SDT multifunctional equipment establishment are critical issues, among others, to be resolved before SDT can be truly translated into clinical use [238, 239].

Besides, lots of nanoparticles designed for the anti-cancer purpose such as GSH-responsive metal–organic-framework (MOF) [240] and magnetic-based nanomaterials [241] have been considered capable of imposing the oxidative stress to transformed cells (Fig. 5). For example, the TBD-Pt(IV)@MOF-199 nanocomposite generated O_2_on light irradiation via consuming intracellular GSH and releasing Pt(IV), leading to the desirable anti-cancer outcome [240]. As another example, the methylene blue immobilized copper ferrite nanocomplex MB-CuFe converted hydrogen peroxide (H_2_O_2_) to ROS and depleted cervical cancer cells by functioning as the photodynamic therapy under 660 nm laser irradiation [241]. Regardless of the intricate design of these nanoparticles, one unanimous concern is the introduction of nanomaterials into the body, where their distribution and retention should be carefully assessed before nanoparticle-based anti-cancer agents can be considered feasible for clinical use.

### Cold atmospheric plasma as an emerging pro-oxidative onco-therapeutics

This section introduces the fourth state of matter, cold atmospheric plasma (CAP), as an innovative type of pro-oxidative strategy in treating cancers (Fig. 5). CAP is composed of a plethora of reactive and oxygen species (RONS) such as short-lived species superoxide anion (O_2_•), OH• and _1_O^2^, and long-lived species such as H_2_O_2_, ozone (O_3_) and nitric oxide (NO), and is typically operated at the atmospheric pressure and the room temperature (37 ~ 44 °C) [1, 242–246]. The diversified biomedical applications of CAP has attracted incremental attention in recent years especially in its use as an emerging onco-therapy [1, 242–247]. Lots of pre-clinical studies have confirmed the use of CAP for treating a large spectrum of cancers including, e.g., breast (especially TNBCs) [243, 248–250], prostate [251], bladder [250], brain [252, 253], colon [254], lung [255] and pancreatic [256, 257] cancers as well as melanoma [258, 259], with diversified molecular mechanisms being proposed. Take breast cancers for example, CAP has been shown capable of inducing TNBC apoptosis and synergize with epithelial growth factor (EGF) for enhanced cancer cell killing [248], halting the epithelial-to-mesenchymal transition process during TNBC cell metastasis [250], uniquely targeting the BCSC cohort of TNBC cells [249], modulating the activity of immune cells in the microenvironment of breast cancer cell [260], and elevating the sensitivity of breast cancer cells to paclitaxel [261, 262], doxorubicin [263], tamoxifen [264]. In addition, the unique benefits of CAP in functioning an emerging onco-therapeutics [265] have been associated with its roles in targeting other critical hallmarks of cancers such as the induction of immunogenic cell death (ICD) among glioblastoma cells [266] and pancreatic cancer cells [267].

The roles of CAP on cancer cells are multifaceted. Yet, all therapeutic benefits of CAP in treating cancers are originated from its intrinsic oxidative nature. It is worth noting that CAP can effectively and uniquely kill transformed cells without harming their healthy peers under appropriate dosage, preventing issues such as therapeutic resistance and evident toxicity.

In addition, CAP can be administrated directly using devices that can action through direct-discharge and indirect-discharge [268, 269]. The source for direct-discharge is called direct dielectric barrier discharge, and devices for indirect-discharge include plasma jets and plasma torches [265]. In general, direct-discharge produces higher amounts of RONS than indirect-discharge. CAP can also be applied indirectly through activating a solution (cell cultivating medium, Ringer’s lactate solution, etc. [270]) to produce plasma-activated medium (PAM) followed by treating cells, animals or patients through injection [271]. Compared with CAP produced from the direct approach that contains ample short-lived species, PAM contain more long-lived species [272, 273]. Such a high plasticity of CAP regarding its production and application makes the clinical translation of the fourth state matter into the field of oncology relatively handy and promising.

## Pro-oxidative approaches in combination with existing intervention strategies

### Pro-oxidative strategies aid in interventional therapy

Tumor resection, utilizing surgical operations to ablate tumors, is one first-line clinical approach for cancer treatment. However, this canonical therapy is challenged by high rates of recurrence that can be primarily attributed to intraoperative cancer cell dissemination and peritumoral invasion [274]. Thus, accurately targeting and eliminating residual tumor cells may prevent metastatic recurrence [275].

Postoperative *in-situ* implantation has been proposed to reduce the likelihood of developing tumor recurrence by removing residual tumor cells. For instance, an implantable sandwich-structured dual-drug reservoir was fabricated that can concomitantly inhibit neoangiogenesis and induce cancer cell death by releasing combretastatin A4 phosphate and tigecycline [276]. Additionally, pro-oxidative strategies can be used for postoperative *in-situ* implantation. For example, a peroxide copper nanoparticles-loaded hydrogel composite was developed that can eliminate residual lesions via inducing cuproptosis [277].

Intraoperative treatment modalities may also be developed to remove residual tumors taking advantages of pro-oxidative approaches. Given that CAP can be prepared in the liquid form (i.e., PAM), PAM can be used to replace sterile solutions containing antibiotics for rinsing during the surgery. PAM has canonically been used for sterilization [278] and wound healing [279], and was reported capable of inducing immunogenic cell death (ICD) [280–284]. Importantly, PAM has been considered as a mild solution for cancer treatment without harming healthy tissues [285]. Thus, it is expected to achieve desirable therapeutic outcome via intraoperative application of PAM during the surgical process. It is also possible to directly expose post-surgical tissues to CAP to remove residual tumor cells during the operation. Dr. Keith Millikan secured the life of a 75-year advanced pancreatic cancer patient in 2016 through the use of Canady Hybrid Plasma™ Scalpel that integrates high frequency monopolar current with CAP to simultaneously cut and coagulate biological tissue [286]. Using this tool, the first clinical trial examining the efficacy and safety of CAP as an onco-therapy was approved by the Food and Drug Administration (FDA) in 2019, where 17 out of 20 stage IV solid tumor patents recruited were still alive by the end of this phase I study in 2021 (NCT04267575).

### Pro-oxidative strategies mitigate adverse effect of chemotherapy

Chemotherapy has been widely accepted as the primary regimen for treating advanced cancers or unresectable metastatic patients [274]. However, chemotherapy also has metastasis-promoting activities by screening out cells with highly invasive phenotypes, expanding cancer stem cell (CSC) cohorts, and activating the EMT process [287]. For instance, the chemotherapeutic agent paclitaxel increased the number of invadopodia, which is one characteristic feature of cancer cells undergoing systemic dissemination [288, 289]; enhanced the amount of circulating tumor cells by over 1000 folds in breast cancer patients [290], the mechanism of which involves hypoxia-inducible factor-1 (HIF1)-dependent p38 mitogen-activated protein kinase (MAPK) signaling axis [291]; and induced EMT by up-regulating Snail and Twist, which are repressive transcription factors of the gene encoding E-cadherin [292].

Chemotherapy functions by killing fast-growing cells without targeting properties. Pro-oxidative strategies may specifically kill cancer cells due to the higher vulnerability of transformed cells to the oxidative stress [1]. This is because that malignant cells have high levels of chaosity that render their anti-oxidative machinery incapable of coping with redox perturbations and thus easily undergo programmed cell death. Therefore, it is plausible to combine chemotherapy with pro-oxidative approaches towards enhanced targeting properties and reduced dosage for minimized adverse effect and low likelihood of promoting metastasis. In addition, pro-oxidative strategies may enhance the therapeutic outcome of chemotherapy. This is because that chemotherapy can enhance cellular oxidative stress [293], rendering its synergies with pro-oxidative approaches naturally compatible. Anthracyclines such as doxorubicin, epirubicin, and daunorubicin, which are widely adopted chemotherapy, have been reported to generate the highest levels of oxidative stress among other agents [294]. It has been reported that anthracycline-based chemotherapy increased levels of oxidative stress and decreased the antioxidant status in small-cell lung cancer patients [295]. Similar results were documented for breast cancer patients after receiving the combined use of doxorubicin, cyclophosphamide and 5-FU [296, 297]; for gastric tumor carriers after receiving adriamyclin, mitomycin and 5-FU; for colon cancer patients having received oxaliplatin, folinic acid and 5-FU; and for prostate cancer patients treated with mitozantrone and prednisolone [298]. A plethora of studies have shown the benefits of combining chemotherapies with pro-oxidative approaches in cancer treatment. For example, CAP, acting as one pro-oxidative tool, synergized with cisplatin in eliminating head and neck cancers [299], and improved the cytotoxic effect of olaparib [300] and paclitaxel [261] in treating TNBC cells.

### Pro-oxidative strategies sensitize metastatic tumors to immunotherapy

Immunotherapy has shown a great promise in resolving tumors. The first checkpoint protein blockade shown effective in cancer immunotherapy was antibody-mediated inhibition of cytotoxic T lymphocyte-associated antigen 4 (CTLA-4) that can inhibit T cell expansion and activation via being translocated to the surface of T cells and competing with CD28 for binding to CD80 and CD86 [301]. More interest has been attracted to design therapeutics targeting the programmed cell death protein 1 (PD1)/ programmed cell death—ligand 1 (PD-L1) axis due to its remarkable treatment efficacy, durable response and mild toxicity, which functions by normalizing instead of purely activating the immune response. Many drugs targeting the PD1/PD-L1 axis have been established and commercialized including anti-PD1 antibodies such as nivolumab (Opdivo), pembrolizumab (Keytruda) and cemiplimab (Libtayo), and anti-PD-L1 antibodies such as avelumab (Bavencio), duravulumab (Imfinzi), and atezolizumab (Tecentriq) [302]. Nivolumab was approved by the FDA for treating advanced melanoma in December 2014, for treating advanced lung cancer in October 2015, for treating unresectable or metastatic melanoma across *BRAF* status in January 2016, for treating locally advanced or metastatic urothelial carcinoma in February 2017, for treating metastatic colorectal cancer in August 2017, for treating completely resected melanoma gained metastasis in December 2017, in treating metastatic or recurrent non-small cell lung cancer in May 2020, for treating advanced renal cell carcinoma in January 2021, for treating unresectable advanced or metastatic esophageal squamous cell carcinoma in May 2022, and for treating unresectable or metastatic urothelial carcinoma in March 2024. Pembrolizumab was approved by the FDA in the treatment of unresectable advanced or metastatic malignant pleural mesothelioma in September 2024. Cemiplimab received its approval for treating advanced cutaneous squamous cell carcinoma in September 2018, for treating advanced basal cell carcinoma and advanced non-small cell lung cancer in February 2021, for treating advanced metastatic castration-resistant prostate cancer in August 2022, and for treating advanced cervical caner in October 2022. Atezolizumab was approved by the FDA in May 2016 as the first PD-L1 inhibitor of urothelial carcinoma, and for treating metastatic non-squamous non-small cell lung cancer in December 2018. Avelumab was approved by the FDA in March 2017 in the treatment of metastatic Merkel cell carcinoma. Duravulumab was approved by the FDA for treating advanced bladder cancer in May 2017, treating unresectable stage III non-small cell lung cancer in February 2018, treating advanced biliary tract cancer in September 2022, treating metastatic non-small cell lung cancer in November 2022, treating advanced or recurrent endometrial cancer in June 2024, and treating limited-stage small cell lung cancer in December 2024.

Though many immunotherapeutic agents targeting CTLA4 or the PD1/PD-L1 axis have been launched into the clinics for cancer treatment, accumulating clinical evidence has suggested that anti-CTLA4 and anti-PD1 are largely futile in eradicating secondary tumors and extending the lifespan of metastatic cancer patients, due to inherent or acquired drug resistance [303]. Consequently, a number of other T-cell checkpoint inhibitors have been established to treat metastatic tumors over the past years, such as inhibitors targeting lymphocyte activation gene 3 (LAG3) and T-cell immunoglobulin and mucin domain 3 (TIM3) [304]. TIM3 expression is associated with advanced cancer stage and lymph node metastasis in lung cancer patients [305], dysfunctional CD8 + T cells and NK cells in patients carrying metastatic melanoma [306, 307]. Importantly, TIM3 is implicated in the adapative resistance of cancer cells to PD1 inhibition. Specifically, TIM3 level increased on CD4 + and CD8 + T cells in metastatic lung cancer patients after anti-PD1 blockage [308]. Indeed, treating lung-tumour-bearing *CC10-rtTA;Tre-egfr*^T790M/L858R^ mice with anti-PD1 and anti-TIM3 extended the lifespan of the diseased animals as compared with applying anti-PD1 blockade alone [308]. In addition, TIM3 inhibition may prime the recruitment of cytotoxic T cells or reinvigoration of exhausted T cells to the tumor site under the metastatic setting. For instance, blocking TIM3 in MMTV-PyMT tumour-carrying mice increased the expression of CXC chemokine ligand 9 (CXCL9) in CD103 + dendritic cells, leading to the recruitment of cytotoxic CXC chemokine receptor 3 (CXCR3) + CD8 + T cells to tumours [309], which is advantageous in treating immunotherapy-naïve or anti-PD1 refractory cancer patients. Being another checkpoint molecule expressed on NK and T cells, increased LAG3 expression was observed on CD4 + and CD8 + T cells among mismatch-repair-proficient colorectal cancer patients developed liver metastasis [310], and was associated with dysfunctional tumour-infiltrating T cells in human metastatic tumours [310–312]. Combinatorial use of anti-PD1 and anti-LAG3 delayed the growth of fibrosarcoma and colorectal cancer, and attenuated the metastasis of ovarian cancer in vivo [313–315].

Despite these pre- and clinical successes as aforementioned, such immune checkpoint therapy only benefits a fraction of patients. An increasing number of researchers have realized the critical roles played by the tumor microenvironment (TME) in the therapeutic response of these treatment regimens. While an immunogenic (hot) TME contain large amounts of tumor infiltrating cells (TILs), cytokines and high level of PD-L1, non-immunogenic (cold) TME exhibit merely no no T cell infiltration and no PD-L1 expression. Therefore, lots of combinatorial strategies have been established to provide improved clinical results with the aim of creating a hot TME by increasing PD-L1 expression, enhancing TIL infiltration, and targeting other type of cells in the TME.

Besides identifying innovative immune targets and establishing concomitant targeting therapeutics against multiple molecules, combining immunotherapy with pro-oxidative approaches may represent another solution to enhance the sensitivity of metastatic cells to immunotherapy. As aforementioned, metastatic cells especially those enriched with CSCs are more vulnerable to pro-oxidative regimen that can easily penetrate through the multi-cell layer within the TME and reach tumor cells. Death of cancer cells in the secondary tumor site elicit signals to the surrounding region to recruit immune cells for enhanced immune cell infiltration and initiate ICD. Indeed, CAP has been shown capable of inducing ICD in a panel of tumor models such as melanoma [280], lung carcinoma [281], nasopharyngeal carinoma [282], and colon cancer [283, 284], restoring skewed macrophage polarization in the TME of TNBC [316], as well as enhancing the therapeutic effect of PD1 blockage therapy in treating head and neck cancer [299] and the treatment outcome of anti-PD-L1 strategy in resolving melanoma [317]. In addition, CAP, being one promising pro-oxidative onco-therapeutic tool, belongs to the naturally existing forms of matter and thus is cheap to produce. It also outweighs other treatment modalities in being specific in killing cancer cells without harming their healthy peers. Thus, combining CAP with immunetherapies may reduce the dosage or treatment time of immunetherapeutic agents, leading to mitigated side effects.

## Conclusion

**Fig. 6:**
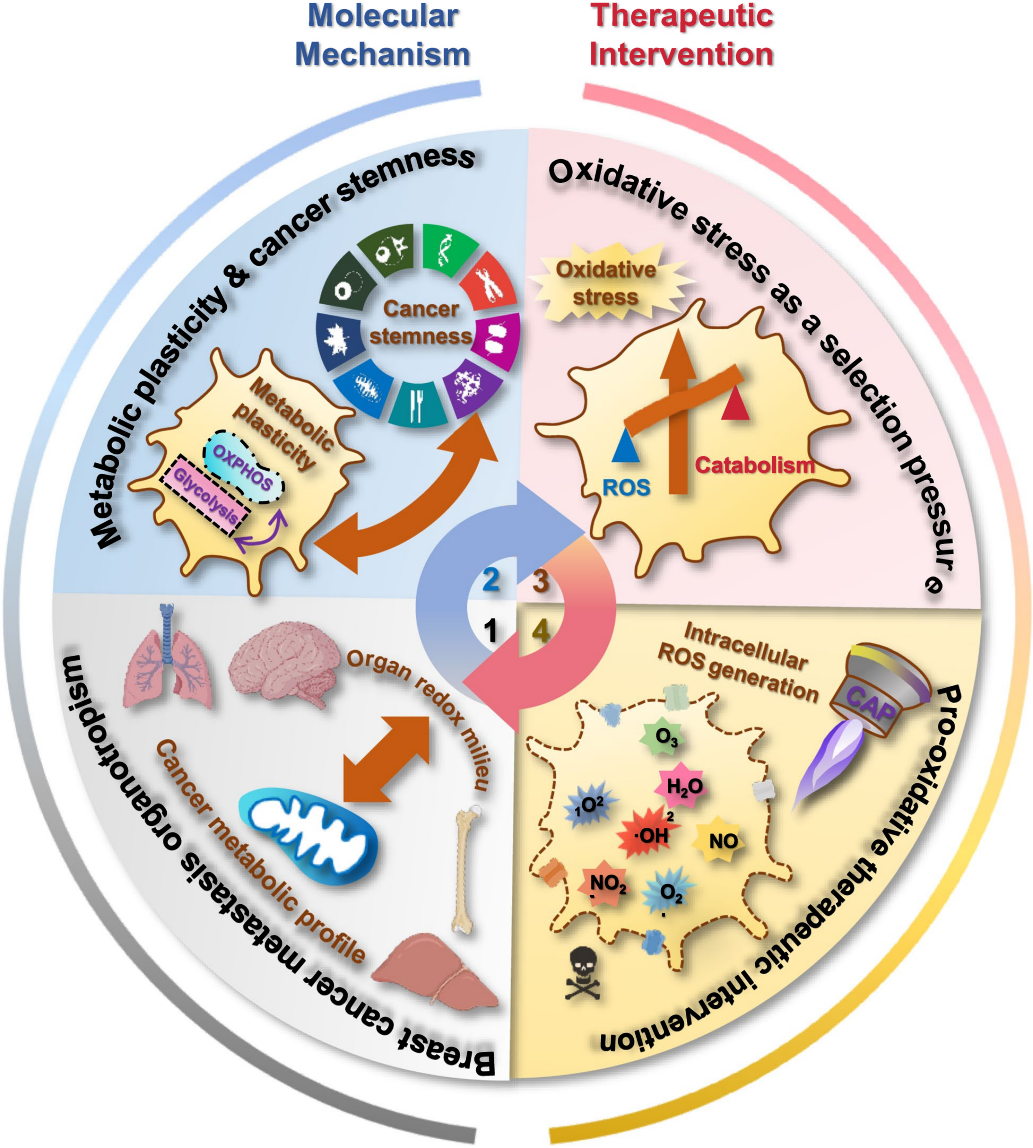
Intrinsic logic for identifying innovative therapeutic approaches from molecular mechanism for cancer metastasis intervention.①Using breast cancer as the tumor model, cancer metabolic profile and organ redox milieu are identified as two primary factors influencing tumor organotropism. ②The metabolic plasticity of cancer cells dictates how much they can adapt to the redox milieu of the secondary lesion, and there exists a positive relationship between the metabolic plasticity and cancer stemness. In other words, the higher metabolic plasticity that cancer cells possess, the more easily they can survive the metastatic process and colonize in the new organ, and the higher cancer stem cell percentage that the metastasized cell cluster contains. ③ During metastasis, cancer cells are subjected to the selection pressure that is imposed by the oxidative stress and fated to happen. Cancer cells capable of increasing catabolism to satisfy the constant high demand of energy supply for traveling and reducing intracelluar ROS generation to counteract with the oxidative stress eventually survive. ④Thus, cancer cells during metastasis are vulnerable to oxidative-stress induced cell death. Yet, therapeutics solely imposing external oxidative stress can only enrich the metastatic cluster with cancer stem cells, approaches capable of inducing intracellular ROS generation can eventually sabotage the anti-oxidant machinery of cancer cells and trigger cell death. In this regard, cold atmospheric plasma (CAP) could be considered as one promising intervention strategy to meet this goal

This paper updated the canonical ‘seed and soil’ theory on metastasis organotropism by viewing the metastatic profile of cancer cells as the ‘seed’ and the redox status of the organ microenvironment as the ‘soil’. Specifically, this paper proposed that cancer cells possessing high plasticity harbor high cancer stemness and can easily colonize in organs with sufficient oxygen supply using breast cancers as the disease model (Fig. 6). From characterizing vital influential factors and the role of redox status in defining organotropism, this paper identified the metastasis process as the most fragile moment sensitive to pro-oxidative perturbations when cancer cells are subjected to the selection of oxidative stress (Fig. 6). Accordingly, this paper reviewed existing pro-oxidative modalities for cancer treatment, and proposed the potential efficacy of CAP, an emerging anti-cancer therapeutics with redox modulatory roles [9, 285], in arresting breast cancer metastasis by intervening with the critical factors identified. In addition to viewing CAP as a redox regulatory tool for interrogating the interactions between cell metabolisms and the oxidative stress, this paper also proposed CAP as a dosage-dependent redox classifier capable of stratifying cancer cells by their metabolic plasticity for specific killing (Fig. 6). These insights may not only advance our understandings on critical factors influencing cancer metastasis organotropism for improved prognosis on cancer metastasis, but also decipher clues why and where pro-oxidative approaches can serve as an oncotherapeutic tool and achieve the optimal treatment outcome.

High metabolic plasticity of cancer cells enables them to easily adapt to the redox environment of the secondary organ site, and thus manifests higher malignancy and metastatic potential. CSCs, a small subset of cancer cells characterized by high differentiation plasticity and self-renewal ability, thus more easily develop metastasis than the bulk tumor cells. Given the giant divergence of the redox status between oxygen-enriched organs such as brain and lung and the tumor initiation loci that is typically subjected to hypoxia, cancer cells metastasized to the brain and lung typically contain higher CSC percentages. As a consequence, cancer cells developed brain or lung metastasis are comparably more difficult to treat than those colonized to other organs [318].

By viewing the metastatic process as an opportunity of interrogating the metabolic profiles of transformed cells when they are at the most fragile moment regarding anabolism and redox sensitivity, it is possible to achieve the aim of ‘survival with tumors’ by killing malignant cells undergoing the metastasis cascade or permanently attracting them to the quiescence state. Yet, cancer cells having survived this selection process may harbor a robust anti-oxidant machinery, resulting in unintended transition of highly-stressed surviving cells into the stem-cell-like state. Thus, though many existing pro-oxidative strategies such as chemotherapy, radiotherapy, magnetic therapy, HVEP, PDT, SDT can effectively ablate cancer cells, they may hardly reduce the rate of cancer relapse and metastasis. The proposed innovative type of pro-oxidative option (i.e., CAP) is unique from this perspective as it can specifically target cancer stem cells [249] and outweighs the other pro-oxidative approaches by, e.g., halting cancer metastasis [250]. These unique traits may be attributed to the multi-modal nature of CAP that modulates the intracellular redox homeostasis instead of simply imposing an external oxidative stress or damaging cells in a brute-force way [319].

CAP is not simply a supplement of current portfolio for cancer therapeutics, but also offers clues for the design of combinatorial anti-cancer strategies by viewing CAP as an adjuvant component. One relevant feature of CAP is to sensitize the TME for enhanced drug sensitivity, rendering it possible to combine CAP with existing therapeutic strategies such as interventional approaches, chemotherapies and immunotherapies to achieve improved treatment response. This may be even more promising for treating cancers with high likelihoods of gaining metastasis or having already gone to the metastasis stage given the essential role of TME and its interplay with metastatic cells in determining cancer cell colonization. Also, with increased research interest in immunotherapies, it is important to resolve issues limiting the clinical efficacy and safety of these treatment approaches. Applying CAP (either directly via ejection or indirectly via administrating its activated liquid) prior to immunotherapies may not only help rewire ‘cold’ tumors back to the ‘hot’ state, but also reduce the chance of developing side effects by reducing the load of immuno-agents; and this may be attributed to the priming role of CAP in the immune system besides its TME-sensitizing capacity.

Another future trend sparked by CAP in designing new approaches for treating cancers is to overcome remaining hurdles limiting the clinical translation of CAP taking advantages of nanomaterials. Despite the plethora of unique beneficial features of CAP identified in oncology, short-lived reactive species within CAP such as OH•, O_2_• and _1_O^2^ play the leading roles in killing transformed cells but have transient life-spans [248, 249]. One strategy would be to preserve the activities of these short-lived species using materials such as covalent organic frameworks, graphene, and hydrogel [320]. Another possibility is to enhance the production of short-lived species by synergizing it with, e.g., magnetic therapies, HVEP, PDT or SDT. For instance, magnetic therapies typically kill cancer cells via the Fenton effect that relies on OH• production, and photosensitizers may serve as the _1_O^2^ producer on light ignite [321, 322]. These offer a plenty of opportunities for cross-disciplinary therapeutic design that may lead one research direction.

Future endeavors may also be devoted to explore therapeutic regimes beyond CAP that are able to take actions via maintaining cellular redox homeostasis, which may open an innovative paradigm for cancer treatment.

## Data Availability

Not available.
